# The current shortage and future surplus of doctors: a projection of the future growth of the Japanese medical workforce

**DOI:** 10.1186/1478-4491-9-14

**Published:** 2011-05-27

**Authors:** Hideaki Takata, Hiroshi Nagata, Hiroki Nogawa, Hiroshi Tanaka

**Affiliations:** 1 Department of Bioinformatics, Tokyo Medical and Dental University, 1-5-45 Yushima, Bunkyo-ku, Tokyo 113-8510, Japan; 2 Faculty of Bioinformatics, Nagahama Institute of Bio-Science and Technology, 1266 Tamura-cho, Nagahama City, Shiga 526-0829, Japan; 3 Japan Medical Information Network Association, Toho Hukasawa Building 5F, 2-2-1 Yushima, Bunkyo-ku, Tokyo 113-8510, Japan; 4 Center of Information in Medicine, Tokyo Medical and Dental University, 1-5-45 Yushima, Bunkyo-ku, Tokyo 113-8510, Japan

## Abstract

**Background:**

Starting in the late 1980s, the Japanese government decreased the number of students accepted into medical school each year in order to reduce healthcare spending. The result of this policy is a serious shortage of doctors in Japan today, which has become a social problem in recent years. In an attempt to solve this problem, the Japanese government decided in 2007 to increase the medical student quota from 7625 to 8848. Furthermore, the Democratic Party of Japan (DPJ), Japan's ruling party after the 2009 election, promised in their manifesto to increase the medical student quota to 1.5 times what it was in 2007, in order to raise the number of medical doctors to more than 3.0 per 1000 persons. It should be noted, however, that this rapid increase in the medical student quota may bring about a serious doctor surplus in the future, especially because the population of Japan is decreasing.

The purpose of this research is to project the future growth of the Japanese medical doctor workforce from 2008 to 2050 and to forecast whether the proposed additional increase in the student quota will cause a doctor surplus.

**Methods:**

Simulation modeling of the Japanese medical workforce.

**Results:**

Even if the additional increase in the medical student quota promised by the DPJ fails, the number of practitioners is projected to increase from 286 699 (2.25 per 1000 persons) in 2008 to 365 533 (over the national numerical goal of 3.0 per 1000) in 2024. The number of practitioners per 1000 persons is projected to further increase to 3.10 in 2025, to 3.71 in 2035, and to 4.69 in 2050. If the additional increase in the medical student quota promised by the DPJ is realized, the total workforce is projected to rise to 392 331 (3.29 per 1000 persons) in 2025, 464 296 (4.20 per 1,000 persons) in 2035, and 545 230 (5.73 per 1000 persons) in 2050.

**Conclusions:**

The plan to increase the medical student quota will bring about a serious doctor surplus in the long run.

## Background

Starting in the late 1980s, the Japanese government decreased the number of students accepted into medical school each year in order to reduce healthcare spending. Student quotas for medical schools were decreased by 7.8% from 1986 to 2006. The resulting shortage of doctors in Japan has inevitably led to deterioration in the quality of care [1,2], and has recently become a serious social problem [3-7].

The per-capita number of medical doctors in Japan is low compared with those in other developed countries. Japan ranks 59th among the World Health Organization's (WHO) 193 member states in terms of number of medical doctors per 1000 persons [8]. The number of medical doctors per 1000 persons in Japan was 2.29 in 2009. This is smaller than the figures for the United States of America (2.56 in 2000) and the United Kingdom (2.30 in 1997). Among the member countries of the Organization for Economic Cooperation and Development (OECD), Japan falls into the category with the fewest doctors per capita, together with Mexico, South Korea and Turkey. The doctor shortage is compounded by Japan's particularly great demand for physicians. Healthcare utilization in Japan is particularly high: the number of consultations per capita is higher in Japan than in any other OECD country [9], and the rates of hospital utilization are high as well. These trends have made the shortage of physicians quite obvious.

In an attempt to solve this problem, the Japanese government decided in 2007 to increase the medical student quota and to maintain it at the new higher level in subsequent years. The dominant party at the time of this decision was the Liberal Democratic Party (LDP); since 2009, however, the ruling party has been the Democratic Party of Japan (DPJ), which has promised to increase the medical student quota 50% more in order to raise the number of medical doctors over 3.0 per 1000 population [10]. The LDP, which is now the largest opposition party, has not announced a specific numerical goal for the Japanese medical workforce [11].

Thus, these two scenarios, that of maintaining the current medical student quota which has been in place since the 2007 increase (LDP), and that of increasing the quota by an additional 50% (DPJ), are recognized as the de facto policies of two major political parties.

**Figure 1:**
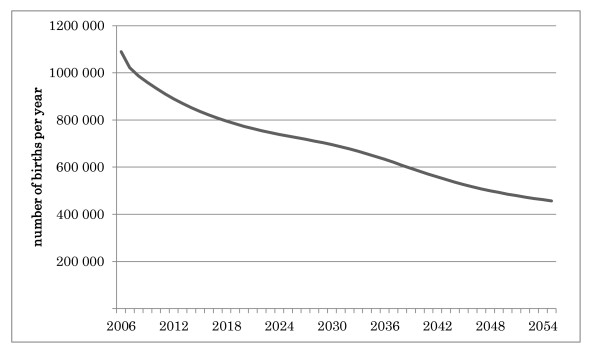
**Projected changes in the total number of births per year in Japan**.

**Figure 2:**
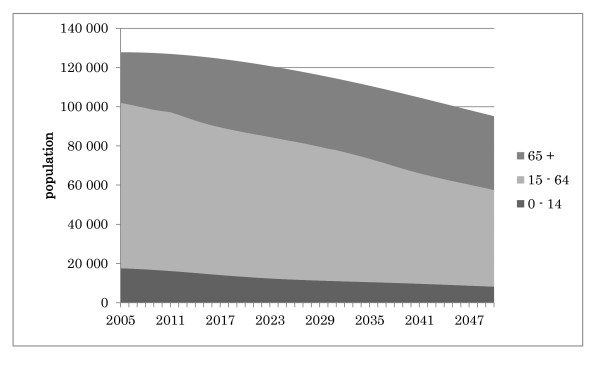
**Projected changes in the Japanese population and age distribution**.

Given that the number of births in Japan per year (Figure 1) and the total population of Japan (Figure 2) are both decreasing [12], this rapid increase in the number of medical students may result in a serious doctor surplus problem, especially after most of the baby boomers die. Yet the Japanese government and the two major parties have given little thought to predicting long-term trends in the supply of and demand for medical practitioners in the debate over the medical student quota.

Our hypothesis is that the proposed additional increase in the medical student quota, in combination with the projected decrease of Japan's total population, will result in a serious doctor surplus in Japan. The purpose of this study is to project the future growth of the Japanese medical workforce and to forecast whether the proposed additional increase in the student quota will cause a doctor surplus. Through computer simulation, we projected the future increase in the number of medical doctors under the following scenarios.

Scenario 1: Maintaining the current medical student quota (8848 per year).

Scenario 2: Increasing the quota by 50%, starting in 2013, as promised by the DPJ.

## Methods

### Modeling the changing population of medical doctors

Our prediction was generated through the following model, which was based on free public data from government and public institutions in Japan. Our baseline year was 2008, and projections were made for the future through 2050.

**Figure 3:**
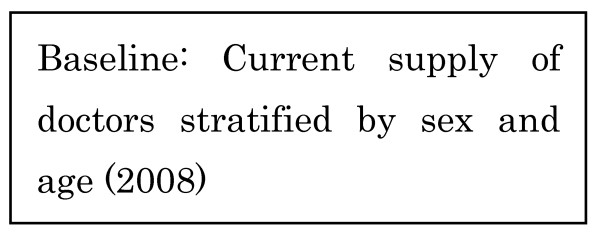
**Baseline: Current number of doctors**.

**Figure 4:**
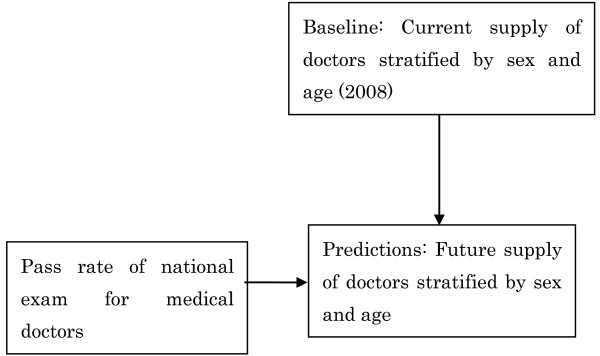
**Addition of estimated number of new doctors**.

1. All medical doctors in Japan are required to report to the government once every two years, providing information about their sex, age, specialty, address, and place of work. These reports are tallied and published as the Survey of Physicians [13]. The number of medical doctors in our baseline year of 2008, stratified by sex and age, was established based on this survey [13] (Figure 3).

2. New medical doctors join the profession every year (Figure 4). In order to become medical doctors in Japan, medical school graduates must pass the national examination for medical doctors. Graduates who do not pass this exam on the first attempt can retake it year after year until they pass. Pass rates for the national examination for medical doctors were assumed to be constant and to be, on average, equal to the average pass rate during the last decade (2000-2009), which was around 90% per year (Table 1) [14].

**Table 1:** Yearly pass rates of national examination for medical doctors

Year	Pass rate	Number of applicants	Number of passers
**2000**	79.1%	8934	7065

**2001**	90.4%	9266	8374

**2002**	90.4%	8719	7881

**2003**	90.3%	8551	7721

**2004**	88.4%	8439	7457

**2005**	89.1%	8495	7568

**2006**	90.0%	8602	7742

**2007**	87.9%	8573	7535

**2008**	90.6%	8535	7733

**2009**	91.0%	8428	7668

**Sum**	88.7%	86542	76744

**Figure 5:**
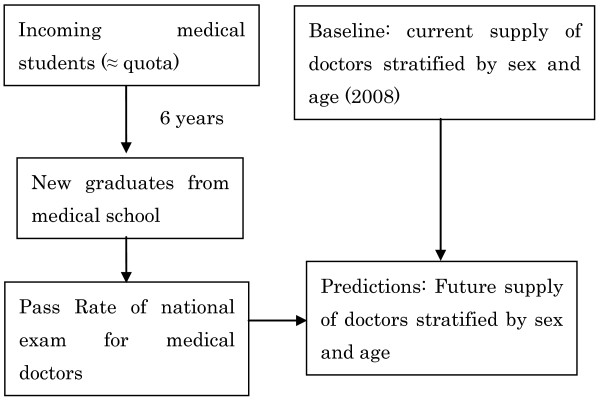
**Most students who enroll in medical school graduate; therefore, the number of graduates taking the national examination every year is approximately equal to the medical student quota that was in place six years earlier**.

3. Because it takes six years to complete medical school in Japan, the number of students graduating from medical schools every year is nearly equal to the medical student quota that was in place six years earlier (Table 2) [14]. As discussed above, the Japanese government controls the number of medical doctors by adjusting the medical student quota (Figure 5). We estimated the number of graduates taking the exam to become medical doctors every year based on the current and proposed quotas. It should be noted that graduates of foreign medical schools can also take the exam to become medical doctors if they pass a screening process administered by the Japanese government, but passing this screening is so difficult that only 20 to 30 graduates of foreign medical schools become doctors in Japan every year; the percentage of new doctors who attended school outside Japan is only about 0.3% per year (Table 3) [14]. For this reason, most students who intend to become medical doctors in Japan attend medical school in Japan. Accordingly, graduates of foreign medical schools were not included in our model.

**Table 2:** Comparison of numbers of graduates and medical student quota six years earlier

Year	Student quota 6 years earlier	Number of Graduates	Graduation Rate
**2000**	7625	7432	97.47%

**2001**	7625	7552	99.04%

**2002**	7625	7831	102.70%

**2003**	7625	7709	101.10%

**2004**	7625	7620	99.93%

**2005**	7625	7545	98.95%

**2006**	7625	7689	100.84%

**2007**	7625	7716	101.19%

**2008**	7625	7519	98.61%

**2009**	7625	7629	100.05%

**sum**	76250	76242	99.99%

**Table 3:** Number of new doctors from foreign medical schools

Year	All new doctors	From foreign schools	Percentage
**2000**	7065	18	0.255%

**2001**	8374	12	0.143%

**2002**	7881	16	0.203%

**2003**	7721	15	0.194%

**2004**	7457	20	0.268%

**2005**	7568	20	0.264%

**2006**	7742	20	0.258%

**2007**	7535	36	0.478%

**2008**	7733	36	0.466%

**2009**	7668	34	0.443%

**sum**	76744	227	0.296%

**Figure 6:**
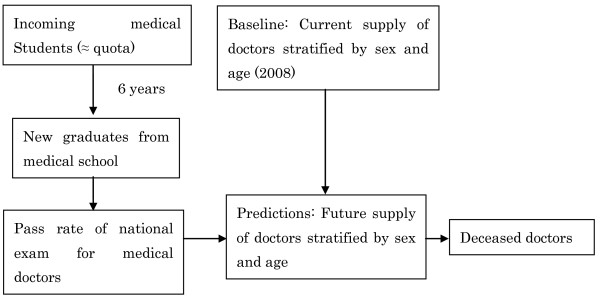
**Subtraction of estimated number of deceased doctors**.

**Figure 7:**
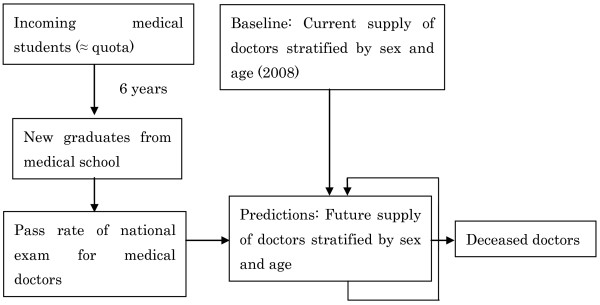
**Repeated addition of new doctors and subtraction of deceased doctors for each year**.

4. The male/female ratio among new medical school graduates was assumed to be constant and, on average, equal to the average ratio during the last decade (2000-2009) [14].

5. Medical doctors were assumed to die in accordance with the death probabilities reported for persons of the same sex and age category in the Complete Life Table (Figure 6) [15].

6. The number of new medical doctors joining the profession was added, and the number of medical doctors dying was subtracted, in two to four steps, for each future year included in the model (Figure 7).

7. Projections concerning future population size were based on the projections published by the National Institute of Population and Social Security Research [12].

This model incorporates data from a wide range of sources which have not previously been drawn together for this type of analysis (Table 4). Other key assumptions are summarized in Table 5. The main outcome measure was the number of medical doctors per 1000 persons.

**Table 4:** Data sources for the simulation model

Variable	Data source
**Current workforce in baseline year (2008)**	The number of physicians reported by the Ministry of Health, Labour and Welfare of Japan (MHLW) in 2008 [13]. Physicians in Japan report to the MHLW every two years, and the MHLW publishes data based on these reports.

**Pass rate for Japanese national examination for medical doctors**	Announcement about national examination for medical doctors (from 94th to 103rd) [14].

**Male/female ratio of new medical graduates**	Announcement about national examination for medical practitioners (from 94th to 103rd) [14].

**The probability that practitioners die**	20th Complete Life Table of Japan published in 2007 by MHLW [15].

**Population projection for Japan**	Population Projection for Japan: 2006-2050 (National Institute of Population and Social Security Research) [12].

**Table 5:** Key assumptions of the base simulation model

Variable	Key assumptions
**New medical graduates**	Only domestic students are counted. The number of medical school graduates is equal to the government's medical student quota.

**The probability that practitioners die**	Practitioners die according to death probabilities calculated using the Complete Life Table.

**Pass rate for Japanese national examination for medical practitioners**	Pass rates remain constant at the rate achieved in the last decade (2000-2009).

**Male/female ratio of new medical graduates**	Male/female ratios of new medical graduates remain constant at the ratio seen in the last decade (2000-2009).

### Simulation scenarios

We used this simulation to project the number of medical doctors under each of the following two scenarios:

Scenario 1: Maintaining the current medical student quota established by the LDP (i.e. 7625 through 2007 and 8848 starting in 2008).

Scenario 2: Increasing the quota by 50% as promised by the DPJ (i.e., 7625 through 2007, 8848 from 2008 to 2012, and 12 000 starting in 2013).

## Results

### Scenario 1: Maintaining the current medical student quota

**Figure 8:**
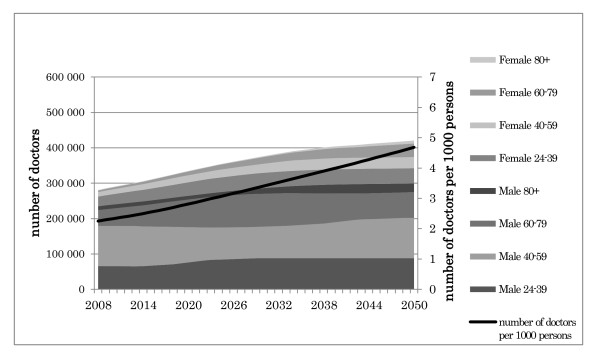
**Outcome of scenario 1: Maintaining the current medical student quota**.

The projected results of Scenario 1 are shown in Figure 8.

In 2008, there were 286 699 doctors in the Japanese medical workforce (2.25 per 1000 persons). Our simulation projected that this figure would reach 365 533 (3.05 per 1000 persons) by 2024. This represents an average annual growth rate of 1.53% per year from 2008 to 2024. Thus, even if the DPJ's proposed additional increase of the medical student quota is not realized, the number of doctors is projected to rise beyond the national numerical goal of 3.0 per 1000 persons in 2024.

After 2024, however, the annual growth rate of the total medical workforce will decrease, but the number of medical doctors per 1000 persons will continue to increase, because the total population will be decreasing. By 2035, there will be 410 999 doctors (3.71 per 1000 persons), and by 2050, there will be 446 050 (4.69 per 1000 persons).

### Scenario 2: Increasing the quota by 50% starting in 2013, as promised by the DPJ

**Figure 9:**
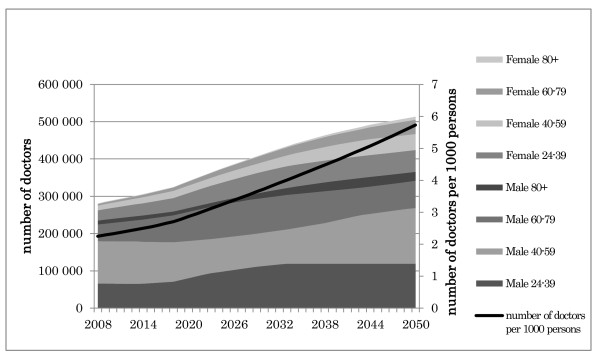
**Outcome of scenario 2: Increasing the medical student quota by 50% starting in 2013**.

The projected results of Scenario 2 are shown in Figure 9.

Our simulation projected that the number of doctors in the Japanese medical workforce would reach 368 196 (3.03 per 1000 persons) by 2022. This represents an average annual growth rate of 1.80% per year from 2008 to 2022. Thus, if the DPJ's proposed additional increase of the medical student quota is realized, the number of doctors is projected to exceed the national numerical goal two years earlier.

After 2022, the number of medical doctors per 1000 persons will continue to increase as the total population decreases. By 2035, the number of medical doctors will reach 464 296 (4.20 per 1000 persons); by 2050, it will reach 545 230 (5.73 per 1000 persons).

### Comparison of the two scenarios

**Figure 10:**
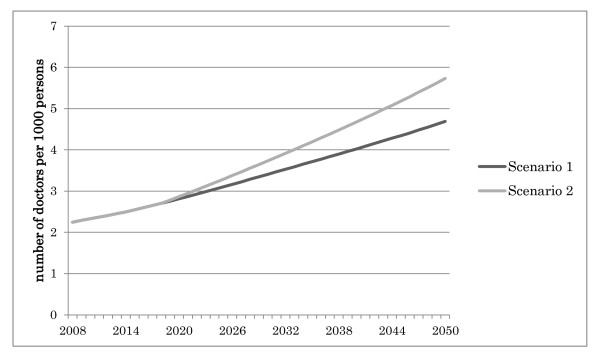
**Comparison of the two scenarios**.

Figure 10 compares the two scenarios' results in terms of the numbers of medical doctors per 1000 persons throughout the projection period.

## Discussion

The Japanese government is currently aiming to adjust the doctor/population ratios to 3.0/1000. Our experience in various medical institutes in Japan allows us to recognize that this target is reasonable. However, whether or not this target level is optimal depends on two elements: the first is future technological breakthroughs in the medical field, and the second is whether or not the Japanese healthcare system, which is based on the medical doctors' monopoly over medical/healthcare treatments, will change.

In many countries, the medical doctors' monopoly over medical treatments has been reviewed, and the functions of paramedical workers have been expanded accordingly [16]. In Japan, however, expanding the functions of paramedical workers in some fields is not as well appreciated as it is in other developed countries because of structural differences [17]. We anticipate that expanding the paramedical functions will not resolve the doctor shortage problem in the near future. This is because the completion of the three essential procedures to expanding paramedical functions will take some time. These three procedures are: 1) reaching consensus regarding this problem, 2) modifying the relevant laws, and 3) educating new paramedical workers in regard to the new functions. We recognize the long-term possibility that some paramedical workers will provide a portion of the medical treatment that doctors currently monopolize. We predict that this possibility will result in a worsening of the doctor surplus in the long run.

Regarding eventual surplus/shortage of other kinds of health workforce, especially nurses, we do not expect a significant change. Some studies have reported a shortage of nurses today [18,19]. However, just as for doctors, demand for them will decrease with a declining population in long term. At present, we did not make predictions for the nurse workforce with our model, as predictions concerning the nurse workforce are difficult using our simple model that predicts workforce supply only from the number of persons acquiring a license. In this way, predictions of nurse workforce numbers are difficult for two reasons: 1) many nurses are not working as nurses even though they possess a license license; 2) the ratio of working nurses to all nurse license holders is strongly influenced by economic conditions [20]. These two reasons cause a gap between number of working nurses and nurse license holders.

The Japanese government is facing a dilemma. The doctor shortage in Japan is currently a serious problem that is hard to solve in the short term, even if the medical student quota is increased. On the other hand, the decreasing population of Japan guarantees that we will eventually face a doctor surplus problem in the long term, even if the medical student quota is not increased.

This means that it is difficult to decide on a medical school quota that would be most appropriate for matching supply and demand of doctors. Moreover, even if we adjust a medical student quota in future to respond to the decreasing population, it can cause an aging problem in the medical workforce: a shortage of young doctors who are generally more adept at coping with new technologies.

Increasing the medical school quota as proposed by the DPJ may diminish the academic performance of the average medical student. Although admission to medical school requires exceptional academic achievement in high school, in the future, more and more students will be able to pass the examination for admission to medical school, because the birthrate in Japan is decreasing. If the medical student quota is maintained at its current level, the percentage of all high school students that qualify for medical school will increase as the population decreases; if the quota is increased, the percentage of qualified students will be even greater. Such a reduction in the level of academic achievement required to become a medical student may reduce the quality of doctors and that of medical treatment.

Furthermore, an increase in the medical student quota may reduce the number of science and engineering students or their average academic performance. Many students who wish to enter medical school are accomplished in science and mathematics; those who do not qualify for medical school often choose to become scientists or engineers instead. If more of the students who are drawn to science and mathematics are able to become doctors, Japan may find itself with fewer or less-qualified scientists and engineers as a result. Therefore the DPJ's proposed increase may be detrimental to the economic potential of Japan in the long term.

Some countries have solved their doctor shortage problems by licensing other types of health practitioners, such as advanced practice nurses, who can fulfill some of the roles of doctors in certain situations. Japan does not offer such licenses, and the political influence of existing professional organizations is so strong that it is impractical and unrealistic to speak of licensing other types of health practitioners.

It will be difficult to resolve this dilemma without the help of foreign countries. In general, a national shortage or surplus of specialists is corrected through international exchange: when a particular specialty is in short supply, specialists are invited into the home country from abroad; in the event of a surplus, the home country's specialists seek work elsewhere. The international exchange of specialists is motivated not by government action but by individual specialists' own desire for better employment.

Most developed countries resolve shortages of health professionals by actively recruiting doctors from other countries. In the 1990s, for example, when the United Kingdom was facing a shortage of doctors, the National Health Service (NHS) actively recruited large numbers of health professionals from abroad, particularly from sub-Saharan Africa, to fill workforce gaps [21,22]. The resulting flow of medical practitioners into the United Kingdom was so large that the recruitment policy was criticized for causing shortages of medical professionals in developing countries [23]. In response to this criticism, the Commonwealth has since introduced guidelines for the recruitment of health workers from abroad [24].

In Japan, however, it is currently more difficult to recruit medical practitioners from abroad because the recognition of foreign licenses is tightly limited, and the number of graduates of foreign schools who are permitted to acquire Japanese licenses is also strictly controlled. We propose that loosening these regulations may reduce the current severe doctor shortage without creating a problematic surplus in the future.

## Conclusions

We conclude that an increase in the medical student quota such as that proposed by the DPJ will not be sufficient to resolve the current doctor shortage and will exacerbate the doctor surplus of the future. It would be more constructive to accelerate the flow of medical doctors from other countries into Japan. We propose that Japan should accelerate the incoming flow of medical practitioners through agreements with other countries permitting early mutual recognition of medical practitioners' licenses, with periodic assessment of source countries to ensure the quality of immigrant doctors. An international comparative study on this matter will be our next research topic.

## Competing interests

All authors declare that they have no competing interests. This paper has not been published elsewhere or submitted for publication to another journal.

## Authors' contributions

All authors designed the study. Hideaki Takata carried out the analyses and drafted several versions of the manuscript. Hiroki Nogawa and Hiroshi Nagata supervised the data analysis. Hiroshi Nagata and Hiroshi Tanaka supervised several versions of the manuscript. All authors read and approved the final manuscript.
